# Treatment of a Suppurating Tooth Socket

**Published:** 1877-01

**Authors:** 


### TREATMENT OF A SUPPURATING TOOTH SOCKET.

The following from a report of the proceedings of the New York Odontological Society, contains some suggestions in reference to the management of some cases, that are well worthy of attention:

"Dr. C. A. Woodward presented a boy twelve years of age, for whom, one year ago, had been extracted the right lower sixth-year molar; from that time to this a large amount of pus had been constantly discharging through a fistulous opening where the tooth was taken from. The gums appeared quite healthy, no inflammation being apparent. On pressing against the chin on the left side (at times when the face is swollen), pus enough to fill the boy's mouth would be discharged.

Dr. W. H. Atkinson. I am always pained to see this kind of a case in the hands of medical men or even of most dentists, for the reason that they have not given sufficiently close attention to such cases to get at the foundation of the trouble, and they fall into a sort of a routine method of management which classifies cases, and then puts them all under one category, for which iodide of potassium, quinine, etc., constitutes the treatment; and that without any direct reference to the objects of the preparation, or to the suiting of the agent to the condition of the body, if it be a tonic. There is so little known about what we mean when we say tonic and stimulant and sedative and all that, that we seem to be under the necessity of going back to the foundation of things and coming up again in our methods, and have a sort of a codification of what is known,—a new and logical arrangement of what we know about the disturbances of function. My idea of a tonic is anything that will supply needed material in the body. Anything that increases or accelerates action in the body, and passes out of the system, is a stimulant; and anything that reduces that action is a sedative. What is the reason that some bodies are cold and others are hot? The reason has never yet been written. I am not sure that I have got the last bottom truth about it myself, but I conceive it to be entirely within the pale of what is called physiological chemistry.

"The tumor in this boy's mouth is a case of that low type of functional aberration that we call strumous. The supply of pabulum, out of which the tissues are fed, was not well elaborated; and then the lad has not been required to use his bodily powers to the limit of his endurance, as every lad should be in order to get the full endowment of his organization. Hence this accumulation took place, but why it located in the chin I don't know. If the boy can be properly fed, it is self-curative; but it will be a very tedious matter, and may leave indurations that can never be removed.

The treatment I would suggest would be to explore the depths and find whether there is one single sinus from the place where the abscess is discharged, or whether there are little lines that run off, sinuses running hither and thither, and also to ascertain whether it is connected with a necrotic gland, that is, a dead gland. Some one of the glands in the chin may have died; and if so, that is a point that will need attention, so as to permit the pabulum that is wept out there for the purpose of healing, to be built into new tissue, and not be poured out to be converted into what is called pus. When you have probed and investigated to the bottom and found out where the trouble is, if necessary, make a counter opening, so that a syringe can wash it out thoroughly.

The constitutional treatment would be iodide of iron, principally; enough quinine to give his blood the right tone, so that it shall be in proper condition to carry the oxygen to the extremities of the vascular circulation.

I would feed him on the best food I could, and give iodide of iron, as much as would digest. You can hardly give too much iodide of iron, because whatever is not appropriated is thrown off; and that, too, is in some sort the case with quinine. The old idea was that quinine was such a terrible thing; but quinine is expensive, and we ought not to waste a particle. The best experiments have shown that no case can ever appropriate more than three grains in a day of the quinine, and that continued four months or more; find out the food he can most easily digest, the richest you can get; aud occasionally give a little potash if the glands show any disturbance."

